# Effects of transparent online open procurement on prices, volumes, and costs of medicines: an interrupted time series study in Ningxia, China

**DOI:** 10.3389/fphar.2024.1362374

**Published:** 2024-08-20

**Authors:** Jingying Gao, Chaojie Liu, Yinming Li, Xizhuo Chen, Tianqin Xue, Yuqing Tang

**Affiliations:** ^1^ School of Medicine and Health Management, Tongji Medical College, Huazhong University of Science and Technology, Wuhan, Hubei, China; ^2^ School of Psychology and Public Health, La Trobe University, Melbourne, VIC, Australia; ^3^ Major Disciplinary Platform Under Double First-Class Initiative for Liberal Arts at Huazhong University of Science and Technology (Research Center for High-Quality Development of Hospitals), Wuhan, Hubei, China; ^4^ Key Research Institute of Humanities and Social Sciences of Hubei Provincial Department of Education, Wuhan, Hubei, China

**Keywords:** interrupted time series analysis, pharmaceutical procurement policy, pharmaceutical expenditures, policy effect, price transparency

## Abstract

**Objectives:**

To assess the effects of the transparent online open procurement arrangement on the prices, volumes, and costs of medicines in Ningxia, China.

**Methods:**

Data were extracted from the Ningxia pharmaceutical procurement platform, covering 16 months of purchase orders (December 2019 to March 2021) prior to the implementation of the transparent online open procurement policy and 20 months of purchase orders after the implementation of the policy (April 2021 to November 2022). Interrupted time series (ITS) analysis was performed to evaluate the effects of the transparent online open procurement policy on the prices, volumes, and total costs of the purchase orders.

**Results:**

After implementation of the transparent online open procurement policy, the average price of purchased medicines showed a declining trend by 0.012 Yuan per month, while the total volume of purchase orders declined at a rate by 1.741 million per month measured by the smallest formulation units and the total costs of the purchase orders decreased at a rate by 5.525 million Yuan per month.

**Conclusion:**

The transparent online open procurement policy resulted in reduced prices, lowered volumes, and lowered total costs of purchased orders of medicines.

## 1 Introduction

According to the most recent statistics provided by the global pharmaceutical information company IQVIA, global expenditure on medicatioxns - defined as the total amount allocated for the procurement of pharmaceuticals from manufacturers, exclusive of off-invoice discounts and rebates -is projected to reach $1.9 trillion by the year 2027 and this expenditure is anticipated to grow steadily at a rate ranging between 3% and 6% annually (IQVIA, 2023). Notably, medications constitute a significant portion of healthcare expenditures in low- and middle-income countries, accounting for 20%–60% of their healthcare spending, in contrast to the 18% observed in countries affiliated with the Organization for Economic Co-operation and Development (OECD) (World Health Organization, 2015). It is crucial to acknowledge that in developing nations, up to 90% of the population rely on out-of-pocket payments for the purchase of medications, resulting in pharmaceuticals being the most substantial household expense, second only to food (World Health Organization, 2015). In China, out-of-pocket payments accounted for 27.0% of its total health expenditure in 2022 (National Health Commission of the People’s Republic of China, 2023b).

China held the position of the world’s second largest consumer in the pharmaceutical sector in 2018, with an impressive expenditure of $137 billion (IQVIA, 2023). The share of China’s consumption of medicines in the world increased from 17.6% in 2015 to 20.3% in 2022 (Fei et al., 2023). Projections suggest that the pharmaceutical market in China is poised for substantial growth, with a volume increase of 8% over the upcoming 5 years (IQVIA, 2023). This surge in demand will be mirrored by a noteworthy 19% increase in pharmaceutical spending, ultimately reaching an estimated range of $140 to $170 billion by the year 2023 (IQVIA, 2023). Notably, from 2010 to 2020, a substantial portion of the total healthcare expenditure, ranging from 30% to 40%, were spent in pharmaceuticals (National Health Commission of the People’s Republic of China, 2023a; Yan et al., 2022). This figure surpassed not only the corresponding percentages observed in the United States (11.0%), Japan (20.7%), and Korea (18.6%) but also exceeded the average of OECD countries (15.9%) (OECD, 2023).

Extensive research has been conducted to investigate the factors contributing to the escalating pharmaceutical expenditure (Main et al., 2022). Pharmaceutical pricing remains a pivotal and predominant factor that significantly contributes to the escalating pharmaceutical expenditure (de Wolf et al., 2005; Kadkhodamanesh et al., 2021; Mirzoev et al., 2021; Akbarpour et al., 2023), gaining increased prominence with each passing year (Ling-hua and Jie-yuan, 2022). Governments worldwide have introduced a range of interventions and policy measures aimed at fostering the rationalisation of drug prices, thereby curtailing pharmaceutical expenses (Lee et al., 2015; Murthy and Okunade, 2016; Hassali and Wong, 2018). These multifaceted strategies encompass diverse pricing mechanisms, as well as practices such as pooled procurement, tendering, and negotiation. These efforts collectively seek to address the challenge of burgeoning pharmaceutical costs (World Health Organization, 2020; Mirzoev et al., 2021). At present, the evidence regarding the effectiveness of these policies in reducing medication prices and enhancing access to pharmaceuticals is notably mixed. These multifaceted considerations underscore the complexity of achieving a balanced and effective pharmaceutical pricing policy (World Health Organization, 2020; Kakkar, 2021).

The fundamental principle of advancing market transparency for pharmaceuticals lies in the public dissemination of information regarding the net prices of healthcare products (World Health Organization, 2019). This concept was initially championed by the 72nd World Health Assembly (WHA), which underscored the critical need for reliable data concerning medicine prices. In this context, the guidelines set forth by the World Health Organization (WHO) on country pharmaceutical pricing policies recommend that nations should actively “Share the net transaction prices of pharmaceutical products with relevant stakeholders, within and external to the country” (World Health Organization, 2020; Riccaboni et al., 2022). The significance of promoting price transparency has been underscored by various initiatives and regulations aimed at enhancing clarity in pricing practices. One notable example is the Medicines Transparency Alliance (MeTA), an initiative led by WHO. This initiative aspired to establish national-level, multi-stakeholder platforms for the sharing of comprehensive data related to the selection, procurement, quality, availability, pricing, promotion, and utilisation of medicines (Paschke et al., 2018). Another pertinent example is the European Union (EU) Transparency Directive, which mandates the public disclosure of list prices for all reimbursable medicines across Europe (European Union, 1988).

Facing the problem of high drug prices and opacity of the drug procurement process in hospitals, in 2000, the Chinese government issued the first policy document on public disclosure of drug price transparency. This retailing price ceilings of drugs set by the government has enabled further regional exploration of drug procurement procedures. In 2005, Sichuan, a western province of China, innovatively piloted centralised drug procurement via the internet and purchasing at the provincial level, with reference to the price ceilings (Jun-feng, 2006). In 2006, Guangdong implemented transparent online open procurement of medicines through price-limit bidding (Jun-he, 2007). In 2009, to further decrease the inflated drug price, the zero-markup for drugs (ZMD) policy was introduced (Ni et al., 2021). In 2015, the milestone national policy document on transparent drug procurement was announced: All localities were encouraged to actively explore and pilot market-oriented drug pricing mechanism instead of strict and static drug retail price cap restriction by government alone. In 2017, the Chinese government issued several policy documents concerning reforming drug procurement and delivery. Standardizations of the Comprehensive National Information Management Platform as well as the Provincial Centralised Drug Procurement Platforms were put forward as core measures to monitor drug prices and supplies. Drug procurement related data, including drug prices were encouraged to be shared with the public. In 2019, the Chinese government issued the “Pilot Plan for National Centralised Drug Procurement”.

The fundamental rationale for promoting price transparency is that it may improves economic efficiency; assists policymakers and researchers with reliable price information; empowers buyers to negotiate more strategically; increases the accountability of manufacturers and governments for prices; and facilitates cost-effective decision-making by prescribers and patients (World Health Organization, 2018; Ahmad et al., 2020). Disclosure and control of drug prices in this study was one of the four aspects of transparency that can occur (World Health Organization, 2018). There was inconsistency in the impact of price transparency on drug prices in current literature. Some studies demonstrated that transparency have brought down the price of medicines, such as a pricing system in the private market conducted in South Africa (Moodley and Suleman, 2019a; Moodley and Suleman 2019b). However, some studies have concluded that transparency has no obvious expenditure saving effect. A previous study in the United Kingdom, for example, found that expenditure on inhaled glucocorticosteroids was unchanged after cost information was provided (Langley et al., 2018). Transparency has even been found to drive prices up, such as a South African study of transparent pricing systems (Bangalee and Suleman, 2016). A systematic review in 2023 concluded that the impact of drug price transparency was still of controversial although important, calling for stronger evidence on aspects as prices, quantites and expenditure (Joosse et al., 2023).

This study was conducted in Ningxia, an underdeveloped province located in northwestern China. In December 2020, Ningxia Public Resources Trading Administration, an agency for administration of governmental related products procurement including pharmaceuticals, announced the notice and rules for transparent online open procurement of medicines (Ningxia Public Resources Trading Administration, 2020a; Ningxia Public Resources Trading Administration, 2020b), with an intention to address the issues of supply shortage of essential medicines by engaging more qualified suppliers and hospitals in free bargaining. It was also expected to bring down transaction costs for both suppliers and hospitals. This study aimed to evaluate the effects of the transparent online open procurement arrangement on the prices, volumes, and total costs of the purchase orders in Ningxia, China. This study was expected to provide evidence of the effectiveness of drug price transparency. Although a previous study conducted in Shanghai, China explored these themes, the evidences were qualitative in nature (Cheng and Bo, 2021). This study used a institutional data with a quasi-experimental design. The findings of this current study were expected to not only address the gap in the literature, but also provide practical reference regarding drug price transparency practice for low and middle income countries similar to China.

## 2 Methods

### 2.1 Study setting

This study was conducted in Ningxia Hui Autonomous Region in northwest China, which covers an area of 66,400 square kilometres with 7.25 million residents (in 2021). It has the largest group of Hui ethnicity. Ningxia is deemed an underdeveloped province in China in terms of both GDP and per capita GDP. In 2021, Ningxia ranked bottom third among the 31 provinces/regions in mainland China in total GDP (452.23 billion Yuan, or $US 63 billion) and 20th in per capita GDP (62,549 Yuan, or $US 8701). Its per capita disposable income reached 38,291 Yuan ($US 5326) for urban and 15,337 Yuan ($US 2133) for rural residents, respectively. In 2021, Ningxia had 4,571 healthcare institutions (including both hospitals and primary care centres), 5.68 inpatient beds per 1,000 population (compared with 6.7 national average), and 8.36 skilled health workers per 1,000 population (compared with 7.97 national average) (Ningxia Hui Autonomous Region Statistics Bureau, 2023; National Health Commission of the People’s Republic of China, 2023a).

### 2.2 Intervention measures

The transparent online open procurement arrangements in Ningxia involve several components.

First, the “Ningxia Pharmaceutical Procurement Platform” (the Platform hereafter) was established by integrating and upgrading the “Ningxia Drug Tendering and Purchasing Platform” and the “Ningxia Centralized Drug Purchasing Network”. The two platforms used to realize the bidding function and the procurement function, respectively. In January 2021, the “Ningxia Pharmaceutical Procurement Platform” overseen by the Ningxia Public Resources Trading Administration was officially put into use and in April 2021, the first batch of drug purchases officially started.

Second, it mandates all qualified suppliers publicly disclosing necessary product information, including the generic names, specifications, dosage forms, and prices of all drugs, to be listed on the official public website on a timely manner. Hospitals are required to procure all of their medicines in line with the product information disclosed on the Platform. The Platform is deemed the only legal channel for public hospitals to make purchase orders.

Third, the Public Resources Trading Administration also coordinates reviews of the product prices negotiated conducted by a group of medical and pharmaceutical experts drawn from the expert database. Those experts will step in for further negotiations for overpriced products. Public hospitals negotiate with the listed suppliers under the supervision of the Public Resources Trading Administration. The price ceiling and average price relating to each of the procured medicines in Ningxia guided the negotiation. After that, medical institutions, on the basis of the listed price of drugs on the platform, combined with their own purchasing volume, bargain with manufacturers through the bargaining function of the Ningxia Pharmaceutical Procurement Platform. Hospitals were allowed to negotiate with listed enterprises on pharmaceutical prices.

Fourth, dynamic price monitoring and adjustment mechanisms were established. Inner reference pricing was used for price adjustment. For enterprises, they are required to actively report the new products prices to Ningxia Public Resources Trading Administration if the prices of the same product listed on other provincial procurement platform was lower. Ningxia Public Resources Trading Administration were then responsible for adjusting the listed price on the platform with reference to the new prices that the enterprises declared. For enterprises that failed to report such information or those with fraud practices, they may be disqualified from the platform. The thorough monitoring and reviewing of pharmaceutical prices are conducted every 6 months.

### 2.3 Study design

This study employed a pre-post design with segmented time series analysis. An interrupted time series is a strong quasi-experimental design in which data are collected at multiple time points before and after the intervention. The advantage of this design is that it can detect a possible underlying secular trend which occurs after the intervention (Koskinen et al., 2015).

The procurement information from December 2019 to November 2022 recorded in Ningxia Pharmaceutical Procurement Platform were extracted for analysis. We aggregated the original data into monthly indicators for the purposed of the study. The basic idea behind the A.M. index system analysis method (Addis A. & Magrini N.’ method) of drug expenditure was that changes in price, volume, and structure were the three main drivers of changes in drug expenditure (Addis and Magrini, 2002). Based on this idea, we chose these variables to explore changes in expenditure, and we did not include structure changes in our study because there was no systematic difference in structure before and after the intervention (Ningxia Public Resources Trading Administration, 2017; Ningxia Public Resources Trading Administration, 2020a). Three indicators were synthesized for analysis: monthly procurement price, volume and cost of medicines. These variables were largely used in current drug cost related studies (Wouters et al., 2017; Dos Santos et al., 2022). Both monthly procurement price and volume were standardized by smallest pack unit (bottle, bag, box, etc.), a commonly used standardization for pharmaceutical price and volume (Wouters et al., 2017). The monthly procurement volume of pharmaceuticals was calculated as sums-up of procurement volumes of each specific product in each month. The monthly procurement price of pharmaceuticals was calculated by dividing total monthly pharmaceutical cost by total monthly procurement volume.

One wave of segmented analysis was conducted in this study. The purchasing information over a 36-month period (from December 2019 to November 2022) were collected. April 2021 (i.e., the 17th month) was chosen as the intervention point for the implementation of pharmaceutical transparent online open procurement policy. This analysis enable us to explore the impact of Ningxia pharmaceutical transparent online open procurement policy on pharmaceutical procurement.

### 2.4 Statistical analysis

The interrupted time series (ITS) was used to analyze the procurement price (CNY), total volume of purchase orders measured by the smallest formulation units (millions of units) and total costs of the purchase orders (CNY in millions), they acted as dependent variables in this study. The linear regression equation is constructed as follows:

$$Y_{t}=\beta_{0}+\beta_{1}\cdot T_{t}+\beta_{2}\cdot I_{t}+\beta_{3}\cdot T\text{ after }I_{t}+\varepsilon_{t}$$

In this model, *Y*_*t*_ is the outcome indicator in month *t*; *T*_*t*_ is a continuous variable indicating the months passed at month *t* since the start of the observation period; *I*_*t*_ represents the two periods before (value = 0) and after (value = 1) the intervention; *T* is a continuous variable indicating months passed since the intervention (time prior to the intervention is coded 0). *β*_*0*_ is the initial level estimate, i.e. the study variable at *t* = 0; *β*_*1*_ is the trend estimate of the change in time variable *t* in the pre-intervention study variable, i.e., the baseline slope estimate; *β*_*2*_ was the level of change in the study variables before and after the intervention; *β*_*3*_ is the trend change of the study variable after the intervention, that is, the difference between the post-intervention slope and the pre-intervention slope; *β*_*1*_+*β*_*3*_ is the slope of the trend of study variables after intervention; *ε*_*t*_ is random error.

The level of autocorrelation in the model was estimated using the Durbin-Watson test (Savin and White, 1977). If autocorrelation exists, the Prais-Winsten method was used to deal with autocorrelation (Turner et al., 2021). All of the analyses were performed using STATA17.0 software (Stata Corp LP, College Station, TX, United States).

## 3 Results

**FIGURE 1:**
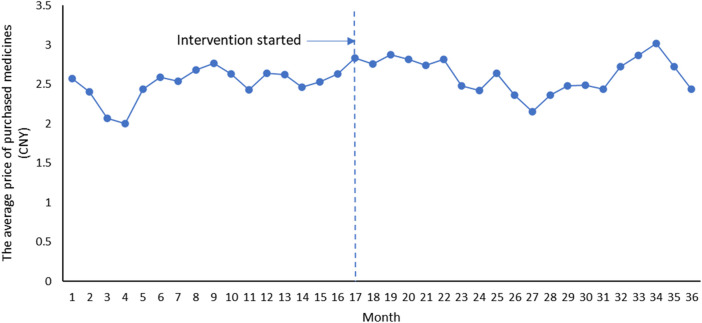
Trend of average price of purchased medicines.

**FIGURE 2:**
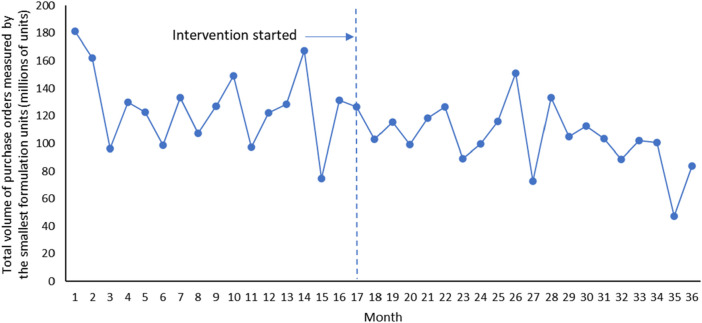
Trends in total volume of purchase orders measured by the smallest formulation units.

**FIGURE 3:**
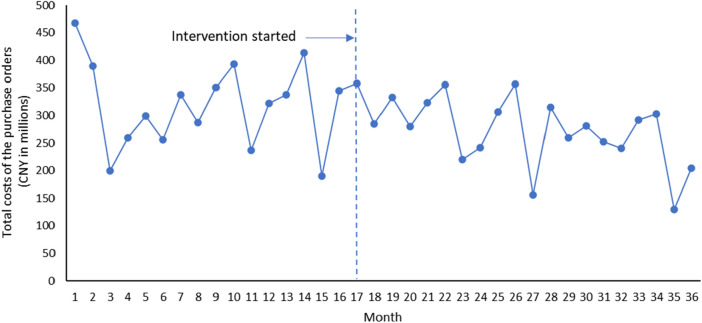
Trends in total costs of the purchase orders.

Overall, it can be seen that after the implementation of Ningxia pharmaceutical transparent online open procurement, the average price of purchased medicines has changed from an upward trend to a downward trend (Figure 1). The downward trend of total volume of purchase orders measured by the smallest formulation units and total costs of the purchase orders has accelerated significantly after the implementation of Ningxia pharmaceutical transparent online open procurement (Figures 2, 3).

ITS analysis shows that before the implementation of the pharmaceutical transparent online open procurement policy, the average price of purchased medicines is increasing at a trend of 0.010 yuan per month [*p* = 0.556, CI = (−0.025, 0.046)]. In the first month of policy intervention, the average price of purchased medicines increased by 0.147 yuan, and the change was not statistically significant [*p* = 0.418, CI = (−0.217, 0.510)]. Compared with the upward trend of 0.010 yuan before policy intervention, the upward trend of the average price of purchased medicines decreased by 0.022 yuan, and the change was not statistically significant [*p* = 0.366, CI = (−0.072, 0.027)], that is, the average price of purchased medicines decreased at 0.012 yuan per month after policy intervention (*β*_*1*_+*β*_*3*_) (Table 1).

**TABLE 1:** Detailed parameters of ITS analysis.

Item	*Coef.*	*p*	[95% *CI*]	
The average price of purchased medicines (CNY)				
Baseline level, *β_0_*	2.427	0.000	2.088	2.766
Baseline trend, *β_1_*	0.010	0.556	−0.025	0.046
Level change, *β_2_*	0.147	0.418	−0.217	0.510
Trend change, *β_3_*	−0.022	0.366	−0.072	0.027
Total volume of purchase orders measured by the smallest formulation units (millions of units)				
Baseline level, *β*_*0*_	138.572	0.000	106.248	170.896
Baseline trend, *β*_*1*_	−1.560	0.352	−4.921	1.802
Level change, *β*_*2*_	7.643	0.595	−21.376	36.662
Trend change, *β*_*3*_	−0.181	0.920	−3.830	3.468
Total costs of the purchase orders (CNY in millions)				
Baseline level, *β*_*0*_	329.908	0.000	225.526	434.289
Baseline trend, *β*_*1*_	−1.596	0.756	−11.954	8.763
Level change, *β*_*2*_	22.980	0.556	−55.691	101.651
Trend change, *β*_*3*_	−3.929	0.474	−14.977	7.119

The total volume of purchase orders was decreasing at a trend of 1.560 million smallest formulation units per month, and the change was not statistically significant [*p* = 0.352, CI = (−4.921, 1.802)]. In the first month of policy intervention, total volume of purchase orders increased by 7.643 million smallest formulation units, with no statistically significant change [*p* = 0.595, CI = (−21.376, 36.662)]. Compared with the downward trend of 1.560 million before the policy intervention, the upward trend of total volume of purchase orders increased by 0.181 million smallest formulation units, and the change was not statistically significant [*p* = 0.920, CI = (−3.830, 3.468)], that is, the total volume of purchase orders after the policy intervention decreased at a trend of 1.741 million smallest formulation units per month (*β*_*1*_+*β*_*3*_) (Table 1).

The total costs of the purchase orders was decreasing at a trend of 1.596 million yuan per month, and the change was not statistically significant [*p* = 0.756, CI = (−11.954, 8.763)]. In the first month of policy intervention, the total costs of the purchase orders increased by 22.980 million yuan, and the change was not statistically significant [*p* = 0.556, CI = (−55.691, 101.651)]. Compared with the downward trend of −1.596 million before the policy intervention, the upward trend of the total costs of the purchase orders decreased by 3.929 million yuan, and the change was not statistically significant [*p* = 0.474, CI = (−14.977, 7.119)], that is, the total costs of the purchase orders after the policy intervention decreased at a monthly trend of 5.525 million yuan (*β*_*1*_+*β*_*3*_) (Table 1).

## 4 Discussion

Our research found that the implementation of Ningxia pharmaceutical transparent online open procurement policy resulted in reduced prices, lowered volumes, and lowered total costs of purchased orders of medicines, and the implementation of this policy can be considered to be quite effective. The average price of purchased medicines changed from an upward trend to a downward trend after the policy intervention, and the downward trend of total volume of purchase orders and total costs of the purchase orders accelerated significantly.

The transparent online open procurement of drugs in Ningxia encompassed some major aspects: First, information on products prices were publicly disclosure under public scrutiny. On the one hand hospitals may benefit with the possibility of expenditure rationalization, and suppliers on the other hand with sales on a larger scale (Sigulem and Zucchi, 2009). Second, a unified platform integration of two separated systems enables unified and simplified government supervision, especially the transaction process by hospitals, manufacturers and deliver enterprises. Comprehensive information may also promote rational procurement decisions by hospitals regarding drug alternatives, etc. Thirdly, public disclosures of prices benefits government regulators for a clearer picture on products prices all over China, leaving more information supporting bargaining with enterprises about their listed prices, largely simplified local price supervision process. Fourth, all qualified drug manufacturers were encouraged to participate in open bidding, competition among suppliers were intensified. Fifth, the openness and transparency of the whole transaction process may also effectively prevent corruptions. From this research, such comprehensive intervention had the potential to bring down drug prices. Cost-savings can also be achieved. The procurement volumes of drugs by hospitals were decreased.

The results of this study are due to a combination of factors, in which transparency may have played a key role in lowering drug prices, but there may have been other elements in the overall policy that played a role, such as upgrading the consolidation of drug purchasing platforms and encouraging companies to bid for medicines, etc., and it is worthwhile to explore further the role played by the specific individual elements. Increasing transparency is one of the solutions proposed by both researcher and practitioners to reduce prices (Franzen et al., 2020). This is especially important for underdeveloped countries where drug corruptions were common. Peru, Zambia, and Jamaica, for example, have promoted the smooth procurement of medicines mainly by strengthening transparent legislation, open procurement process and related information transparency (World Health Organization. Regional Office for the Eastern Mediterranean, 2009). Simply public disclosure of pharmaceutical prices on the internet would make a great positive impact (Paschke et al., 2018). It was also believed that increased manufacturer transparency would improve the overall fairness and efficiency of price negotiation system (Moon, 2018; Colbert et al., 2020), leading to price reduction on both originator and generic medicines. Previous studies in China have shown that transparency of information on the procurement of essential medicines reduces the number of high-priced medicines procured, which is conducive to promoting the accessibility of essential medicines and the rational use of medicines (Xin et al., 2013).

Information technology in public contracting helps creating a more competitive, transparent, and accountable procurement system. Ukraine, for example, introduced a centralized e-procurement system that is known as ProZorro (Law of Ukraine, 2015). Public dissemination about the procurement process combined with an electronic bidding system is considered an efficient strategy for price competition and also an anti-corruption tool (Cohen and Montoya, 2001). Example of how transparency improved procurement processes is also found in Chile. It’s pharmaceutical procurement system CENABAST had historically been inefficient and non-transparent. Empirical analysis showed that savings from use of the reformed system were estimated to be between 5% and 7%, with demonstrated price reductions (Cohen and Montoya, 2001; Singer et al., 2009). In 2013 their efforts reportedly increased transparency in 180 hospitals (Navarrete, 2014). Merely publishing purchase prices may be insufficient to reduce prices. Full bidding and fulfillment via electronic means are key enabling factors for medication price reduction. This has been explored by several Brazilian studies. A 2009 study of an e-procurement system introduced to nine Brazilian hospitals revealed a decrease in the price over ten percent (Sigulem and Zucchi, 2009). While such decrease can’t be attributable to transparency alone, volume purchasing arrangement may also play a crucial part (Sigulem and Zucchi, 2009). Price reductions may be attributable to greater purchasing leverage, in addition to improved transparency. Later on, another study in 2015 verified such hypothesis that an Internet-based strategy to improve pricing transparency did not lead to statistically significant reductions in actual purchase price, suggesting that merely publishing purchase prices for medications may be insufficient to reduce prices (Kohler et al., 2015).

In addition to pharmaceutical transparency, many benefits have been found in other areas of transparent online, some studies have emphasized the economic and social significance of adopting online pharmacies (Al Halbusi, 2024), finding that social media use and technological turbulence in SMEs can have a positive impact on business performance (Al Halbusi, 2022a; Hassani et al., 2022), as well as on e-entrepreneurial intentions (Al Halbusi, 2022a; Al Halbusi, 2022b). In addition, the adoption of knowledge management systems and artificial intelligence can improve business sustainability (Al Halbusi, 2023).

The results of the study can be better understood when considering the unique context of Ningxia. Ningxia, was an autonomous region in China, with a distinctive economic and medical background. Additionally, the technological upgrades of the Ningxia pharmaceutical procurement platform facilitated government supervision and drug transactions, which also played a crucial role in observing the results. As stated in the study setting in the Methods section, Ningxia as an economically and medically underdeveloped province needs efficient spending, which aligns with the goals of the transparent online open procurement policy. The observed reduction in medicine prices and costs is reflective of the region’s need to optimize limited resources while maintaining access to essential medicines.

This study suffered from major limitations. First, we only had access to institutional data of Ningxia, the generality of the results may be limited since nationwide data would be more applicable. The data we got was the average price of purchased medicines, the total volume of purchase orders, and the total costs of the purchase orders for each month of transparent online open procurement, and we got synthetic data rather than original data. Second, when dealing with drug prices and volumes, Defined Daily Dose (DDD) standardization would have be more concise, but since our access to the detailed data was restricted, the smallest package unit were used as an alternative. In addition, this study lacked control variables, data to adjust for drug type were not available, and administrative costs associated with the procurement process were not considered. Ningxia, as a less developed provincial level autonomous region, faced with similar problems as those undeveloped countries, including insufficient medical resources and government revenue. The demand for pharmaceuticals was also low with limited population. Thus, even with these flaws, this study makes significant contribution to the international readers especially for developing countries facing with souring pharmaceutical expenditure issues and medical corruption issues. This study evaluated the policy effects, provided new evidence that expands public understanding of the impact of transparent online open procurement, may provide valuable lessons for health policy decision makers elsewhere.

## 5 Conclusion

Ningxia pharmaceutical transparent online open procurement policy was characterized by a high transparency on pharmaceutical prices. The whole transaction process between hospitals, manufacturers and delivery enterprises were under strict government scrutiny. Public disclosure of pharmaceutical prices enabled an inner reference price adjustment. From our empirical assessment, this comprehensive procurement strategy resulted in reduced prices, lowered volumes, and lowered total costs of purchased orders of medicines. While the effect of such comprehensive reform on patient outcome or drug access needs further investigation.

## Data Availability

The data that support the findings of this study are available from Ningxia Public Resources Trading Administration but restrictions apply to the availability of these data, which were used under license for the current study, and so are not publicly available. Data are however available from the authors upon reasonable request and with permission of Ningxia Public Resources Trading Administration.
